# Estimating genomic breeding values from the QTL-MAS Workshop Data using a single SNP and haplotype/IBD approach

**DOI:** 10.1186/1753-6561-3-s1-s10

**Published:** 2009-02-23

**Authors:** Mario PL Calus, Sander PW de Roos, Roel F Veerkamp

**Affiliations:** 1 Animal Breeding and Genomics Centre, Animal Sciences Group, Wageningen University and Research Centre, P. O. Box 65, 8200 AB Lelystad, The Netherlands; 2 CRV, PO Box 454, 6800 AL Arnhem, The Netherlands

## Abstract

Genomic breeding values were estimated using a Gibbs sampler that avoided the use of the Metropolis-Hastings step as implemented in the BayesB model of Meuwissen *et al., Genetics *2001, 157:1819–1829.

Two models that estimated genomic estimated breeding values (EBVs) were applied: one used constructed haplotypes (based on alleles of 20 markers) and IBD matrices, another used single SNP regression. Both models were applied with or without polygenic effect. A fifth model included only polygenic effects and no genomic information.

The models needed to estimate 366,959 effects for the haplotype/IBD approach, but only 11,850 effects for the single SNP approach. The four genomic models identified 11 to 14 regions that had a posterior QTL probability >0.1. Accuracies of genomic selection breeding values for animals in generations 4–6 ranged from 0.84 to 0.87 (haplotype/IBD vs. SNP).

It can be concluded that including a polygenic effect in the genomic model had no effect on the accuracy of the total EBVs or prediction of the QTL positions. The SNP model yielded slightly higher accuracies for the total EBVs, while both models were able to detect nearly all QTL that explained at least 0.5% of the total phenotypic variance.

## Background

The applied models to estimate genomic breeding values described in this paper, are derived from a multiple QTL mapping model described by Meuwissen and Goddard [1]. The methods are implemented using variable (i.e. in this case presence of a QTL or not on a putative QTL position) selection via Gibbs sampling [2]. Thus, the applied Bayesian method avoids the computationally costly Metropolis-Hastings step that was implemented in the BayesB model of Meuwissen *et al. *[3].

## Methods

### Parameterization of the model

The data were analyzed with five different models considering only additive genetic effects. The first model (called 'HAP_POL') was:

$$y_{i}=\mu +s_{i}+u_{i}+\sum_{j=1}^{5994}\left(q_{ij1}+q_{ij2}\right)\;v_{j}+e_{i}$$

where *y*_*i *_is the phenotype of animal *i*, *μ *is the overall mean, *s*_*i *_is a fixed effect for sexe, *u*_*i *_is the polygenic effect of animal *i*, *v*_*j *_is the direction of the QTL effects of the haplotypes at putative QTL position *j*, *q*_*ij*1 _(*q*_*ij*2_) is the size of the QTL effect for the paternal (maternal) haplotype of animal *i *at putative QTL position *j*, and *e*_*i *_is the residual term for animal *i *[1]. Note that the total effect of a haplotype is modeled as *q*_*ij. *_× *v*_*j*_, and that *q*_*ij*_. and *v*_*j *_may have a positive or negative value. The covariance among polygenic effects (*u*.) was modeled as **A **× $\sigma_{\text{G}}^{\text{2}}$, where A is the relationship matrix which was based on the full pedigree and $\sigma_{\text{G}}^{\text{2}}$ is the polygenic variance. The second model (called 'HAP_NOPOL') was the same as HAP_POL, but omitted the polygenic component. The HAP models assumed a putative QTL in the midpoint of each marker bracket. The covariances among haplotypes at bracket *j *(*q*_.*j*._) were modeled as **H**_*j*_, which is the matrix of estimated IBD probabilities among the haplotypes at the midpoint of bracket *j*. The variance of *q*_.*j *._was assumed 1, while *v*_*j *_is a scale parameter that accommodates for a bracket to have a large (small) effect, if a QTL is (not) present. IBD probabilities between haplotypes were calculated using the algorithm of Meuwissen and Goddard [4], which combines linkage disequilibrium with linkage information and, for each bracket *j*, considers 20 surrounding markers and all available pedigree information. The effective population size was assumed 100 and the number of generations since an arbitrary founder population was also assumed 100, as in Meuwissen and Goddard [1]. All pairs of base haplotypes (i.e haplotypes of first generation of genotyped animals) with an IBD probability above 0.95 were clustered, using a hierarchical clustering algorithm. If the matrix of IBD probabilities among base haplotypes was not positive definite after clustering, the matrix was bended by adding |*min_eigenval*| + 0.01 to all the diagonal elements, where |*min_eigenval*| is the absolute value of the lowest (negative) eigenvalue. The matrix was subsequently inverted by LU denomposition. The elements in $H_{j}^{-1}$ for the descendant haplotypes were then calculated using the algorithm of Fernando and Grossman (1989) [5]. When the IBD probability of descendant haplotypes with one of their parental haplotypes exceeded 0.95, the descendant haplotype was clustered with this parental haplotype.

The third applied model called 'SNP_POL' was:

$$y_{i}=\mu +s_{i}+u_{i}+\sum_{j=1}^{6000}\left(q_{ij1}+q_{ij2}\right)\;v_{j}+e_{i}$$

where *y*_*i*_, *s*_*i*_, *μ*, and *u*_*i *_are as in model 1, *v*_*j *_is the direction of the effects of the alleles at marker locus *j*, *q*_*ij*1 _and *q*_*ij*2 _are the sizes of the marker effects of animal *i *at marker locus *j*, and *e*_*i *_is the residual term for animal *i*. The fourth model (called 'SNP_NOPOL') was the same as SNP_POL, but omitted the polygenic component.

For reasons of comparison, a fifth model was applied, which did include the polygenic effects, but omitted the SNP effects. This model was called 'POL'.

### Solving algorithm

For all models, a Markov chain Monte Carlo method using Gibbs sampling was used to obtain posterior estimates for all the effects in the model [1]. The scale parameter of a putative QTL at locus *j*, *v*_*j*_, was sampled from a normal distribution N(0, $\sigma_{\text{V}}^{\text{2}}$), if a QTL was present in bracket *j*, whereas *v*_*j *_was sampled from N(0, $\sigma_{\text{V}}^{\text{2}}$/100) if no QTL was not present in bracket *j*. The variance of *v*_*j*_, $\sigma_{\text{V}}^{\text{2}}$, was sampled from an scaled inverse chi-square distribution with a prior variance of 0.058. This prior variance was calculated as the additive genetic variance, estimated using model 'POL', divided by 30, i.e. assuming 30 additive and unrelated QTL affecting the trait, across the 6 chromosomes. The presence of a QTL in bracket *j *was sampled from a Bernoulli distribution with probability equal to $\frac{\text{P}(v_{j}|\sigma_{\text{V}}^{\text{2}})\times {\mathrm{Pr}}_{\text{j}}}{\text{P}(v_{j}|\sigma_{\text{V}}^{\text{2}})\times {\mathrm{Pr}}_{\text{j}}+\text{P}(v_{j}|\sigma_{\text{V}}^{\text{2}}/100)\times (1-{\mathrm{Pr}}_{j})}$, where P(*v*_*j*_|$\sigma_{\text{V}}^{\text{2}}$) is the probability of sampling *v*_*j *_from N(0, $\sigma_{\text{V}}^{\text{2}}$), i.e. $\frac{1}{\sqrt{2\pi \sigma_{\text{V}}^{\text{2}}}}{\text{e}}^{-\frac{v_{j}^{2}}{2\sigma_{\text{V}}^{\text{2}}}}$, and Pr_j _is prior probability of the presence of a QTL in bracket *j*. Pr_j _was calculated per bracket as five times (i.e. assuming five QTL per chromosome) the length of bracket *j*, divided by the total length of all the brackets on the chromosome. More details on the prior distributions and the fully conditional distributions can be found in Meuwissen and Goddard [1]. The Gibbs sampler was implemented using residual updating, which was proven to be an computationally efficient way to solve the equations [6]. The Gibbs sampler was run for all models for 30,000 iterations and 3,000 iterations were removed as burn-in.

## Results

Estimates for the mean and both sexes were small in the SNP and HAP models, i.e. the estimates ranged from 7.3E-05 to 3.2E-02 (results not shown). Accuracy of estimated breeding values, i.e. the correlation with the simulated breeding values, were calculated for all five models for animals without phenotypic information (Table 1). The accuracy of estimated breeding values was similar across the four genomic models. However, the accuracy of the HAP models decreased across generations, while the accuracy of the SNP models appeared to be constant across generations. Coefficients of the regression of true breeding values on estimated breeding values were 0.85–0.86 for the HAP models and 0.94–0.96 for the SNP models, indicating that the bias of the estimated breeding values was larger for the HAP models than for the SNP models. The correlations among estimated breeding values (EBVs) of animals with phenotypes and the correlation with their phenotypes were calculated (Table 2). The correlation between phenotypes and EBVs was largest for the POL model, while both for the SNP and HAP models it was slightly higher when the polygenic effect was included, compared to when it was excluded. The correlations among EBVs of animals without phenotypes were also calculated (Table 3). Correlations among EBVs from the SNP and HAP models were all > 0.94, indicating small differences in predictive ability between those models. The correlation between the POL and the other models were much lower for animals without phenotypes (0.21–0.23; Table 3), compared to animals with phenotypes (0.76–0.78; Table 2).

**Table 1:** Correlations (reflecting accuracy) between true and estimated breeding values, and coefficients of regression of true breeding values on estimated breeding values (estimated using all five models) for animals without phenotypes in generations 4, 5 and 6.

**Method (Group)**	**Gen-4**			**Gen-5**			**Gen-6**			**Gen-4–6**		
	**Corr.**	**b**	**r2**	**Corr.**	**b**	**r2**	**Corr.**	**b**	**r2**	**Corr.**	**b**	**r2**

HAP_POL (B1)	0.87	0.872	0.75	0.83	0.850	0.70	0.81	0.807	0.66	0.84	0.854	0.70
HAP_NOPOL (B2)	0.87	0.882	0.76	0.84	0.863	0.71	0.81	0.801	0.66	0.84	0.859	0.71
SNP_POL (B3)	0.86	0.893	0.73	0.87	0.969	0.75	0.86	0.942	0.74	0.86	0.943	0.74
SNP_NOPOL (B4)	0.87	0.910	0.76	0.87	0.982	0.76	0.87	0.958	0.76	0.87	0.958	0.75
POL (B5)	0.26	0.435	0.07	0.05	0.104	0.002	0.15	0.452	0.02	0.07	0.143	0.01

**Table 2:** Correlations between phenotypes and estimated breeding values for animals with phenotypes, estimated using all five models.

**Model**	**HAP_POL**	**HAP_NOPOL**	**SNP_POL**	**SNP_NOPOL**	**POL**
Phenotype	0.639	0.627	0.625	0.615	0.799
HAP_POL		0.998	0.989	0.988	0.782
HAP_NOPOL			0.991	0.990	0.771
SNP_POL				0.997	0.770
SNP_NOPOL					0.762

**Table 3:** Correlations between estimated breeding values for animals without phenotypes, estimated using all five models.

**Model**	**HAP_NOPOL**	**SNP_POL**	**SNP_NOPOL**	**POL**
HAP_POL	0.993	0.941	0.942	0.209
HAP_NOPOL		0.946	0.949	0.205
SNP_POL			0.994	0.230
SNP_NOPOL				0.228

**Figure 1:**
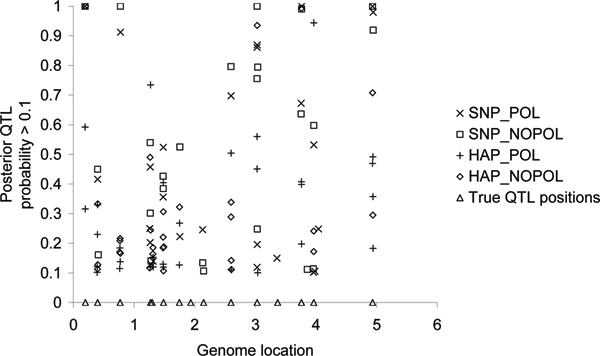
**Posterior QTL probabilities along the genome, estimated using HAP_POL, HAP_NOPOL, SNP_POL, and SNP_NOPOL, and the position of QTL that explained > 0.5% van de phenotypic variance**.

Posterior QTL probabilities > 0.1 were plotted along the genome for all genomic models (Figure 1). All models were able to detect nearly all QTL that explained at least 0.5% of the total phenotypic variance.

## Discussion

The presented methods have been applied in multiple studies, where they proved to be able to detect QTL [1,7] as well as estimate genomic breeding values accurately [7-9]. In the present study, differences in accuracies of the EBVs of the HAP and SNP models were small, which is in agreement with the finding that for r^2 ^values between adjacent markers of ~0.2 the differences in accuracies of the HAP and SNP models are negligible [8]. Apparently, including linkage analysis information next to linkage disequilibrium information in the model (i.e. going from the SNP to the HAP model), does not yield additional information to estimate effects more accurately.

Interestingly, the POL model yielded a higher correlation between EBV and phenotype than the genomic models. However, the accuracy of the EBVs for animals with the genomic models were 0.93–0.94, while the accuracy for the same animals were only 0.70 for the POL model (results not shown).

## Conclusion

For the provided data set, including a polygenic effect in the genomic model had no effect on the accuracy of the total EBVs or prediction of the QTL positions. The SNP model yielded slightly higher accuracies for the total EBVs, while both models were able to detect nearly all QTL that explained at least 0.5% of the total phenotypic variance.

## Competing interests

The authors declare that they have no competing interests.

## Authors' contributions

MPLC carried out the analyses and drafted the manuscript. APWR helped to draft the manuscript and to interpret the results. RFV helped to interpret the results and present them in the manuscript. All authors read and approved the final manuscript.
